# Co-administration of 1,25-dihydroxyvitamin D3 and infliximab improves colitis in mice by modulating Treg differentiation

**DOI:** 10.22038/IJBMS.2024.74640.16209

**Published:** 2024

**Authors:** Yan Hu, Yang Wang, Ying Chen, ChuanYing Li, Yun Long, Cheng Wu

**Affiliations:** 1 Department of Pediatrics, The First Affiliated Hospital of Anhui Medical University, 218 Jixi Road, Hefei, China, 230022; 2 Department of Gastroenterology, Anhui Children’s Hospital, 39 Wangjiang East road, Hefei, China, 230051

**Keywords:** Colitis, Infliximab, Interleukin-10, Regulatory T cells, Vitamin D

## Abstract

**Objective(s):**

The combination of TNF-α inhibitors and vitamin D in colitis remains to be elucidated. In the present study, we revealed the benefit of infliximab (IFX) and vitamin D in a mouse model of Ulcerative colitis (UC).

**Materials and Methods:**

A dextran sulfate sodium-induced colitis model was used. The therapeutic effect of the combination was evaluated by symptom and histopathology analysis. The synergistic mechanism was explored by detecting the regulatory effect of the combined therapy on Regulatory T cell (Treg) differentiation.

**Results:**

IFX and 1,25-dihydroxyvitamin D3 (VitD3) synergistically prevented the development of colitis by improving clinical signs, pathological and hematological manifestation, and inhibiting intestinal inflammation (decreasing TNF-α, IL-1β, and IL-6). Co-administration of IFX (2.5 mg/kg) with VitD3 or IFX (5.0 mg/kg) with VitD3 was more effective than administration of IFX (2.5 mg/kg, 5.0 mg/kg). There was no difference in therapeutic effect between IFX (5.0 mg/kg) and VitD3+ IFX (2.5 mg/kg) groups or between the VitD3+IFX (5.0 mg/kg) and VitD3+ Azathioprine (AZA) groups. VitD3 or combination therapy showed more powerful regulation of splenetic Treg differentiation and IL-10 production than IFX alone. Moreover, VitD3 alone or in combination induced higher levels of Foxp3 and IL-10 than IFX in colon tissue. In ulcerative colitis patients, serum VitD3 levels positively correlated with Treg levels.

**Conclusion:**

VitD3 and IFX synergistically inhibit colitis based on their powerful regulation of Treg differentiation. VitD3 combined with IFX is an alternative therapy for patients who are intolerant to standard doses of IFX or combination of IFX and AZA.

## Introduction

Ulcerative colitis (UC) is a nonspecific inflammatory disease of the colon. UC is characterized by diarrhea with discharge of mucus and blood, cramping abdominal pain, and inflammation and edema of the mucous membrane with patches of ulceration. In the past decade, UC has become a global public health problem, with increased prevalence of UC around the world, incurable by current medicines, and high recurrence rates. Presently, the clinical goal of therapy is to induce and maintain clinical and endoscopic remission (1, 2). Although the precise etiology of UC is still unclear, aberrant immune responses, including dysfunctional immune cells and imbalanced inflammatory cytokine networks, are widely thought to underlie UC formation (1, 2). Tumor necrosis factor-alpha (TNF-α) was the first cytokine identified as a target for the treatment of inflammatory diseases. It is well accepted that TNF-α plays a key role in initiating and amplifying inflammatory autoimmune diseases (3), such as UC, rheumatoid arthritis, Crohn’s disease (CD), ankylosing spondylitis, psoriasis, and psoriatic arthritis. Therefore, blockade of TNF-α action by monoclonal antibodies has been successfully developed to prevent UC (4). Infliximab (IFX) is a chimeric monoclonal antibody that binds soluble and membrane-bound TNF-α and is used to induce and maintain clinical remission (5). However, some patients are not sensitive to IFX therapy at the beginning of treatment, or some initially sensitive patients lose their drug sensitivity at the middle and late stages of treatment, leading to UC recurrence (6, 7). Therefore, to obtain a better therapeutic effect, IFX has been used together with other immunosuppressants, such as thiopurines (8). However, severe side effects induced by the combination frequently occur, such as infection and malignant tumors (9).

Vitamin D is a fat-soluble vitamin required for normal growth of teeth and bones and is produced in general by ultraviolet irradiation of sterols found in milk, fish, and eggs. Vitamin D is recognized as a regulator of both innate and adaptive immune responses, and vitamin D deficiency has been associated with the development of several immune-mediated disorders, including inflammatory bowel disease (IBD), CD, and UC (10-11). 1,25-Dihydroxyvitamin D3 (VitD3) is the main active form of vitamin D in the body and exerts powerful pharmacologic effects by binding to its receptor. VitD3 plays a protective role in UC by maintaining the integrity of mucosal homeostasis and the healing capacity of the colon epithelium (12). A previous study suggested that vitamin D is an important adjuvant therapy for the treatment of UC (12, 13). However, the study was not sufficiently powered to evaluate the benefit of combination therapy with IFX+VitD3 in UC. Therefore, in this study, we investigated the effect of co-administration of VitD3 and IFX for treatment in a dextran sulfate sodium (DSS)-induced Ulcerative colitis model and evaluated the benefits.

## Materials and Methods

***Participants***

This study was approved by the ethics committee of the Anhui Provincial Children’s Hospital, and all procedures involving human participants were performed following the ethical standards described in the 1964 Declaration of Helsinki and its later amendments. From January 2019 to December 2021, a total of 7 patients (four boys and three girls, mean age: 10.28 years) and 9 healthy controls were enrolled in the current study. All patients conformed to the Third European Evidence-based Consensus on Diagnosis and Management of UC (14). Each subject underwent detailed clinical evaluation and endoscopic assessment. Colonoscopy was performed to assess the extent of the disease and endoscopic severity, and the severity of UC was determined on the basis of the disease activity index (DAI) score, in which 0–2 was classified as remission, 3–6 as mild disease, 7–10 as moderate disease, and 11–12 as severe disease. The following inclusion criteria were used: (1) diagnosed UC based on clinical records, colonoscopy, and pathology; (2) no history of chronic inflammatory diseases (such as diabetes); (3) no history of large gastrointestinal surgery; and (4) no history of use of medications such as TNF-α blockers, nonsteroidal anti-inflammatory drugs, vitamin D supplementation, and immunosuppressants within the past three months. All patients and healthy control participants signed written consent prior to their enrollment in this survey. Serum samples from participants were prepared at the time of enrollment, and VitD3 and TNF-α were detected using enzyme-linked immunosorbent assay (ELISA) kits (Catalog# DY410-05, RDKAP1971, R&D Systems, Minneapolis, MN, USA). Moreover, fecal samples were collected, and calprotectin levels were detected using a kit according to the manufacturer’s instructions (Catalog# DS8900, R&D Systems, Minneapolis, MN, USA).

***Animals***

C57BL/6 male mice (8 weeks old) were purchased from SLAC Laboratory Animal Co., Ltd. (Shanghai, China). Animals were fed under a specific pathogen-free environment at 24 ±1 °C, 40–75% relative humidity, and a 12:12 hr light/dark cycle. All mice were provided with food and tap water *ad libitum* for one week to acclimate before experiments. The present animal study was approved by the ethics committee of Anhui Medical University, and all procedures were performed in accordance with ethical standards described in the guidelines of laboratory animal use and care of the European Community (EEC Directive of 1986; 86/609/EEC).

***UC induction and treatment***

UC was induced in the mice by a regular method reported previously (15). In brief, the mice were provided with sterilized tap water with 3% DSS (Catalog# D806297, Macklin Biochemical Technology Co., Ltd) in the drinking water for 7 days to induce colitis. The water was changed every other day. Moreover, the normal control mice were given sterilized tap water without 3% DSS. One week later, 3% DSS-treated mice were randomly divided into seven groups (eight mice per group): (1) normal group; (2) model group; (3) VitD3 group (2 μg/kg, p.o, Catalog# D1530, Sigma‒Aldrich, St. Louis, MO, USA); (4-5) IFX group (Catalog# HY-P9970, MedChemExpress LLC, Shanghai, China, 2.5 mg/kg, 5.0 mg/kg, IP); (6) VitD3+ IFX group (2.5 mg/kg); (7) VitD3+IFX group (5.0 mg/kg); (8) Azathioprine (AZA, 10 mg/kg, p.o, Catalog# A4638, Sigma‒Aldrich, St. Louis, MO, USA)+IFX group (5.0 mg/kg). The mice were orally administered VitD3 once a day for 14 days after 3% DSS treatment. Moreover, the animals also received IFX by intraperitoneal injection at 4 and 11 days after 3% DSS treatment. The mice in the normal and DSS model groups received normal saline only. On day 14, all animals were sacrificed, and tissue collection was conducted for further use.

***Assessment of UC severity***

During the study period, DAI was assessed by loss of weight, stool consistency, and presence of blood in the stool. The DAI was evaluated by the researcher who was blinded to the group information. DAI values were recorded daily. Percentage of weight loss (A): 0 meant no weight loss; 1 meant a 0–5% loss; 2 meant a 5–10% loss; 3 meant a 10–15% loss; and 4 meant over 15% loss. Fecal states (B): 0 meant normal feces; 1–2 meant wet and soft feces; and 3–4 meant loose feces. Severity of hematochezia (C): 0 was normal or occult blood; 1–2 was dim blood; and 3–4 was obvious blood. The DAI score was calculated according to (A+B+C)/3.

***Assessment of hematological manifestation***

At the end of the study, blood samples were collected from each animal. Hematological parameters, such as red blood cell (RBC) counts, hematocrit (HCT), hemoglobin (HGB), monocytes, and total white blood cell (WBC) counts, were measured by an IDEXX ProCyte DX hematology analyzer (IDEXX, Westbrook, ME, USA).

***Histopathological analysis***

Colonic histopathology was detected under a light microscope. For pathological assessment, the colonic specimens were fixed in 4% formalin, embedded in paraffin, cut into 5 μm thick sections, and then stained with hematoxylin and eosin (H&E). Pathological damage was evaluated according to previously described criteria (16).

***Cytokine measurement***

At the end of the study, blood was collected, and serum was prepared to measure inflammatory cytokine levels. Moreover, colon tissues were collected and homogenized and then centrifuged at 2,500×g for 10 min at 4 °C. The supernatant was prepared and then used to detect the levels of interleukin (IL)-1β, IL-6, IL-10, and TNF-α using ELISA kits according to the manufacturer’s protocols (Catalog# MLB00C, M6000B-1, M1000B-1, MTA00B, R&D systems, Minneapolis, MN, USA).

***Flow cytometry analysis***

To analyze the level of regulatory T cells (Tregs) from patients and experimental animals, flow cytometry analysis was performed. Spleen tissues were removed, cut into pieces, ground into suspension carefully, and filtered using a cell strainer. Peripheral blood mononuclear cells (PBMCs) were collected from clinical participants based on the density gradient centrifugation method. PBMCs and splenocytes were fixed and stained with anti-CD4, anti-CD25, and anti-Forkhead Box P3 (Foxp3) antibodies (Catalog# PA5-85858, PA5-116978, PA1-46126, eBioscience, Santiago, USA) and analyzed by a FACSCanto II Flow Cytometer (BD Biosciences, USA).

***Immunohistochemical staining***

Fixed colonic samples were cut into 4-μm-thick sections, deparaffinized and rehydrated through a series of xylene and ethanol washes. Sections were blocked with 1% goat serum albumin for 10 min at room temperature and incubated with a primary antibody against Foxp3 (Catalog# ab215206, Abcam, Cambridge, UK) overnight at 4 ℃. After incubation with the secondary antibody, 3,3-N- diaminobenzidine (Catalog# P0203, Beyotime Biotech. Inc., Shanghai, China) was added to each section for 30 sec. The sections were observed using an Olympus AX70 microscope and quantitatively analyzed by Image-Pro Plus 6.0 software (Media Cybernetics, Inc., Rockville, VA, USA).

***Statistical analysis***

The data were analyzed using the SPSS 16.0 statistical package. Multiple comparisons were performed by one-way analysis of variance (ANOVA) followed by an LSD t-test. A value of P<0.05 was considered statistically significant, and all results are presented as the mean ± SD.

## Results

***VitD3 positively correlated with Treg levels in UC patients***

**Figure 1:**
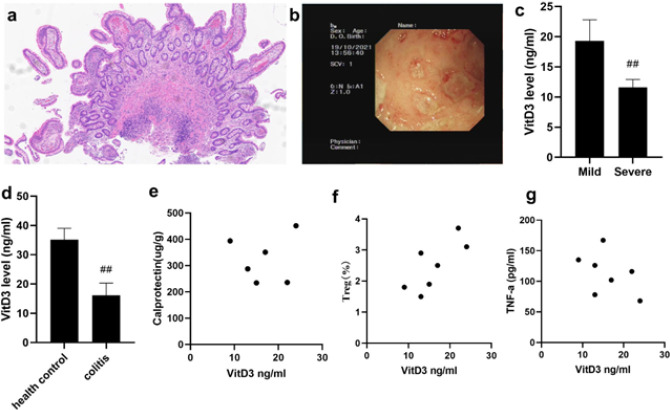
VitD3 correlated with Treg levels in UC patients

To characterize the status of VitD3 in UC patients, we detected the level of serum VitD3 and then analyzed its relationship with inflammation-related molecules in UC patients (Figure 1). A total of 7 UC patients were included, and their median age was 10.29 years. As shown in Figure 1c-1d, the median level of VitD3 in mild patients was significantly higher than that in severe patients (*P*<0.01). Moreover, the level of VitD3 in UC patients was significantly lower than that in the healthy control group (*P*<0.01). VitD3 levels positively correlated with Tregs (*P*<0.05, Figure 1f) but not with TNF-α and calprotectin levels in UC patients (Figure 1e, 1g).

***VitD3 and IFX synergistically improved colitis symptoms in mice***

**Figure 2:**
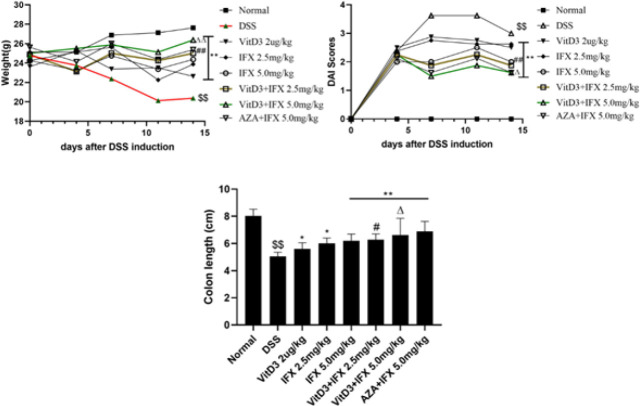
VitD3 and IFX synergistically improved the clinical manifestation of colitis in mice

To explore the therapeutic effect of the combination of VitD3 and IFX, we evaluated the changes in body weight, DAI score, and colon length of mice in each group. As shown in Figure 2, treatment of mice with VitD3, IFX (2.5 mg/kg; 5.0 mg/kg), VitD3+IFX (2.5 mg/kg; 5.0 mg/kg) or AZA+IFX effectively inhibited the development of DSS-induced colitis by increasing average weight, reducing DAI scores, and improving the lowered colon (P<0.01). Compared with IFX (2.5 mg/kg or 5.0 mg/kg), the inhibitory effect of VitD3+IFX (2.5 mg/kg or 5.0 mg/kg) on DSS-induced clinical signs was more powerful (P<0.01). Moreover, there was no significant difference in the colitis symptoms of mice that received IFX (5.0 mg/kg) and VitD3+IFX (2.5 mg/kg). Moreover, there was no significant difference in the efficacy between VitD3+IFX (5.0 mg/kg) and AZA+IFX(5.0 mg/kg).

***VitD3 and IFX synergistically improved the hematological parameters of colitis mice***

**Figure 3:**
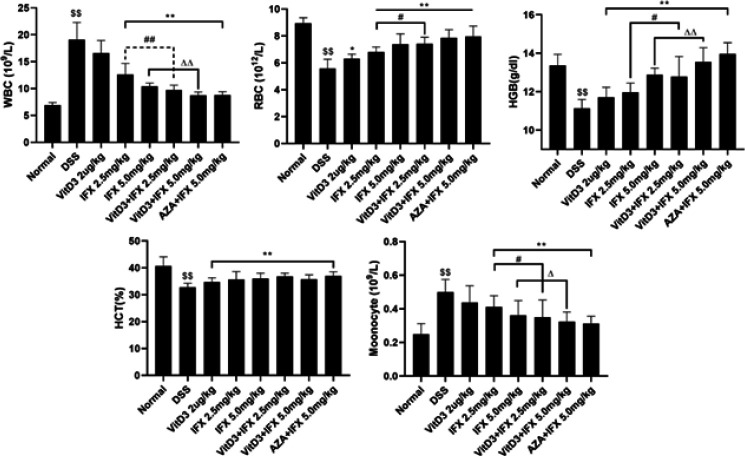
VitD3 and IFX synergistically improved the hematological parameters of colitis mice

Hematological parameters, such as RBCs and WBCs, are important laboratory indices to assist in the diagnosis of colitis. As shown in Figure 3, compared with those of the normal group, the levels of RBCs, HGB, and HCT were significantly reduced, and WBC and monocyte levels were significantly increased in the DSS group (*P*<0.01). After administration of IFX (2.5 mg/kg; 5.0 mg/kg), VitD3+IFX (2.5 mg/kg; 5.0 mg/kg), or AZA+IFX (5.0 mg/kg), the levels of WBCs and monocytes were significantly decreased compared with those in the DSS group, and the levels of RBCs, HGB, and HCT were significantly increased compared with those in the DSS group (*P*<0.01). Moreover, VitD3 treatment showed little regulatory effect on these parameters. Further analysis was conducted between different medicine groups. We found that the regulatory effects of VitD3+IFX (2.5 mg/kg) on present hematological parameters (excerpt for HCT) were more powerful than those of IFX (2.5 mg/kg) (*P*<0.05). A similar result was observed when comparing VitD3+IFX (5.0 mg/kg) and IFX (5.0 mg/kg) (*P*<0.05). Interestingly, there were no significant differences in these parameters between VitD3+ IFX (2.5 mg/kg) and IFX (5.0 mg/kg) or between VitD3+IFX (5.0 mg/kg) and AZA+IFX (5.0 mg/kg).

***VitD3 and IFX synergistically improved the pathological manifestation of colitis mice***

**Figure 4:**
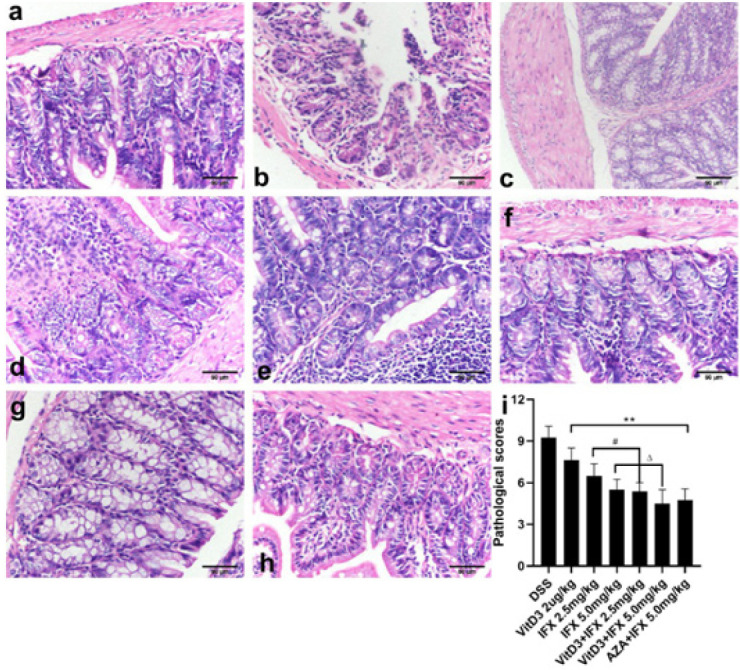
VitD3 and IFX synergistically improved the pathological manifestation of colitis in mice

As shown in Figure 4b, DSS-administered mice showed striking hyperemia, infiltration of inflammatory cells into the mucosa, and exfoliation of goblet cells and epithelial cells. These histological features did not appear in the normal group (Figure 4a). Treatment of mice with VitD3, IFX (2.5 mg/kg; 5.0 mg/kg), VitD3+ IFX (2.5 mg/kg; 5.0 mg/kg) or AZA+IFX (5.0 mg/kg) effectively improved mucosal damage and inflammatory cell infiltration (Figure 4c-4 h). We further quantitatively analyzed the histopathology of colon tissues in each group. As shown in Figure 4i, intervention with different medicines effectively reduced the scores of DSS-treated mice (*P*<0.01). Moreover, the score in the VitD3+ IFX (2.5 mg/kg) group was significantly reduced compared with that in the IFX (2.5 mg/kg) group (*P*<0.05). A similar result was also observed comparing the score between VitD3+IFX (5.0 mg/kg) and IFX (5.0 mg/kg) (*P*<0.05). Finally, there were no significant differences in pathological scores between VitD3+IFX (2.5 mg/kg) and IFX (5.0 mg/kg) or between VitD3+IFX (5.0 mg/kg) and AZA+IFX (5.0 mg/kg).

***VitD3 and IFX synergistically repressed the inflammatory response in colitis mice***

**Figure 5:**
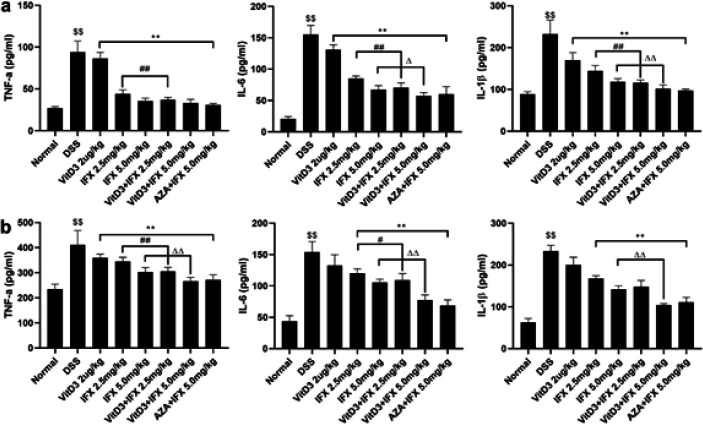
VitD3 and IFX synergistically reduced inflammatory cytokines in colitis mice

To evaluate the anti-inflammatory effect of the combination of VitD3 and IFX, we detected inflammatory cytokines in serum and colon tissue. ELISA results revealed that the levels of the cytokines TNF-α, IL-1β, and IL-6 in the DSS group were significantly increased compared with those in the normal group (Figure 5a-5b, *P*<0.01). After administration of IFX (2.5 mg/kg; 5.0 mg/kg), VitD3+IFX (2.5 mg/kg; 5.0 mg/kg), and AZA+IFX (5.0 mg/kg), serum and colonic levels of TNF-α, IL-1β, and IL-6 were significantly decreased compared with those in the DSS group (Figure 5a-5b, *P*<0.01), while VitD3 treatment alone showed little effect on these cytokines. Moreover, the levels of TNF-α, IL-1β, and IL-6 in the VitD3+IFX (2.5 mg/kg) group were significantly decreased compared with those in the IFX (2.5 mg/kg) group (*P*<0.05). A similar result was observed in the comparison of the score between VitD3+IFX (5.0 mg/kg) and IFX (5.0 mg/kg) (*P*<0.05). Interestingly, there were no significant differences in the cytokine levels between VitD3+IFX (2.5 mg/kg) and IFX (5.0 mg/kg) or between VitD3+IFX (5.0 mg/kg) and AZA+IFX (5.0 mg/kg).

***VitD3 and IFX synergistically regulate Tregs in the spleens of colitis mice***

**Figure 6:**
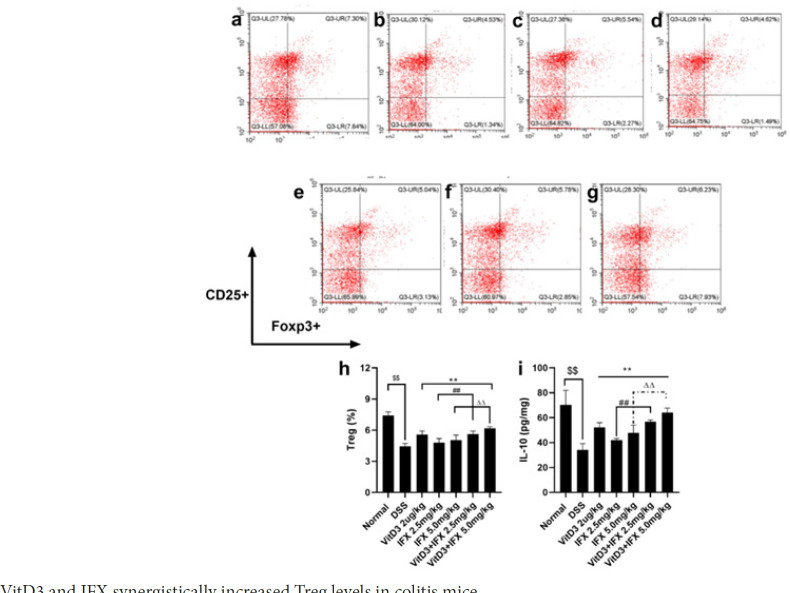
VitD3 and IFX synergistically increased Treg levels in colitis mice

Tregs play a negative regulatory role in the occurrence and development of colitis (17). We continued to investigate the regulatory effect of the combined administration on Tregs in DSS-treated mice. As shown in Figure 6, DSS-treated mice showed decreased levels of Tregs compared with the normal group (*P*<0.01). However, the frequency of Tregs in mice treated with VitD3, IFX (2.5 mg/kg; 5.0 mg/kg), and VitD3+IFX (2.5 mg/kg; 5.0 mg/kg) was 1.25-fold, 1.07-fold, 1.13-fold; 1.26-fold and 1.38-fold greater than that in DSS mice, respectively (*P*<0.01). Meanwhile, the level of Tregs in the VitD3+IFX (2.5 mg/kg) group was significantly increased compared with that in the IFX (2.5 mg/kg) group (*P*<0.01). A similar result was presented in the comparison of Tregs between VitD3+IFX (5.0 mg/kg) and IFX (5.0 mg/kg) (*P*<0.01). Interestingly, there were no significant differences in Treg levels between the VitD3+IFX (2.5 mg/kg) and IFX (5.0 mg/kg) groups. Additionally, IL-10 is the main cytokine secreted by Tregs. We further analyzed the expression of IL-10 secreted by splenocytes. The results are shown in Figure 6i. In brief, compared with the normal group, the level of IL-10 was down-regulated in DSS-treated mice (*P*<0.01). After treatment with VitD3, IFX, or their combination, the expression of IL-10 was significantly increased in comparison with that of the DSS group (*P*<0.01). Moreover, VitD3 and IFX cotreatment synergistically increased IL-10 levels in the spleens of DSS-treated mice.

***VitD3 and IFX synergistically regulate Foxp3+ cells in the colonic tissue of colitis mice***

**Figure 7:**
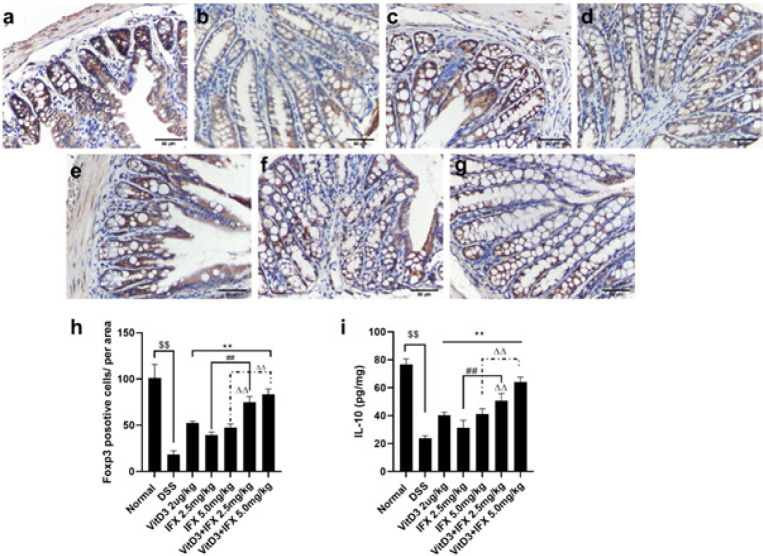
VitD3 and IFX synergistically increased Foxp3 levels in colitis mice

Foxp3 is required for the generation of functional Tregs. We continued to investigate the regulatory effect of the combined administration on Foxp3 levels in DSS-treated mice (18). As shown in Figure 7, DSS-treated mice showed decreased levels of Foxp3 expression compared with the normal group (*P*<0.01). However, the levels of Foxp3 in mice treated with VitD3, IFX (2.5 mg/kg; 5.0 mg/kg), and VitD3+IFX (2.5 mg/kg; 5.0 mg/kg) were significantly increased compared with those in the DSS group (*P*<0.01). Moreover, the expression of Foxp3 in the VitD3+IFX (2.5 mg/kg) group was significantly higher than that in the IFX (2.5 mg/kg) group. A similar result was presented when comparing Foxp3 between VitD3+ IFX (5.0 mg/kg) and IFX (5.0 mg/kg) (*P*<0.01). Interestingly, there was a significant difference in Foxp3 levels between VitD3+IFX (2.5 mg/kg) and IFX (5.0 mg/kg). Additionally, IL-10 is responsible for maintaining the expression and function of Foxp3 in Tregs. We further analyzed the expression of IL-10 in colonic tissues. Compared with the normal group, the level of IL-10 was decreased in DSS-treated mice (*P*<0.01). After treatment with VitD3, IFX, or their combination, the expression of IL-10 was significantly increased compared with that in the DSS group (*P*<0.01). Moreover, VitD3 and IFX cotreatment synergistically increased IL-10 levels in the colonic tissues of DSS-treated mice (*P*<0.01).

a-b. Representative pathological picture and endoscope image of ulcer tissues; c. VitD3 level in mild and severe UC patients; d. VitD3 level in healthy controls and UC patients; e. Correlation between calprotectin and VitD3; f. Correlation between Tregs and VitD3; g. Correlation between TNF-α and VitD3; data are represented as the mean±standard deviation; n=7; ##*P*<0.05 versus severe or colitis group

UC: Ulcerative colitis; VitD3: 1,25-dihydroxyvitamin D3

Data are represented as mean±standard deviation; n=8 per group; $$*P*<0.01 versus normal group; *P<0.05, **P<0.01 versus DSS group; #*P*<0.05, ##*P*<0.01 versus IFX group (2.5 mg/kg); ^∆^*P*<0.05, ^∆∆^*P*<0.01 versus IFX group (5.0 mg/kg).

IFX: Infliximab; DSS: Dextran sodium sulfate; D3: 1,25-dihydroxyvitamin; AZA: Azathioprine

Hematological parameters were determined from the whole blood of mice, and data are represented as the mean±standard deviation; n=8 per group; ^$$^*P*<0.01 versus normal group; ^**^P<0.01 versus DSS group; ^#^*P*<0.05, ^##P^<0.01 versus IFX group (2.5 mg/kg); ^∆^*P*<0.05, ^∆∆^*P*<0.01 versus IFX group (5.0 mg/kg).

IFX: Infliximab; DSS: Dextran sodium sulfate; VitD3: 1,25-dihydroxyvitamin D3; AZA: Azathioprine

(200×, H&E staining) (a) normal group; (b) DSS group; (c) VitD3 group; (d) IFX (2.5 mg/kg); (e) IFX (5.0 mg/kg); (f) VitD3+IFX (2.5 mg/kg); (g) VitD3+IFX (5.0 mg/kg); (h) AZA+IFX (5.0 mg/kg); (i) pathological scores; data are represented as the mean±standard deviation; n=8 per group; **P<0.01 versus DSS group; #*P*<0.05 versus IFX group (2.5 mg/kg); ^∆^*P*<0.05 versus IFX group (5.0 mg/kg)

IFX: Infliximab; DSS: Dextran sodium sulfate; VitD3: 1,25-dihydroxyvitamin D3; AZA: Azathioprine

a: Inflammatory cytokines TNF-α, IL-6, and IL-1β in serum were examined by ELISA; b: TNF-α, IL-6, and IL-1β in colonic tissues were examined by ELISA; data are represented as the mean±standard deviation; n=8 per group; $$P<0.01 versus normal group; **P<0.01 versus DSS group; #*P*<0.05, ##*P*<0.01 versus IFX group (2.5 mg/kg); ^∆^*P*<0.05, ^∆∆^*P*<0.01 versus IFX group (5.0 mg/kg).

IFX: Infliximab; DSS: Dextran sodium sulfate; VitD3: 1,25-dihydroxyvitamin D3; AZA: Azathioprine

Representative flow cytometric dot plot and proportion of Tregs (h) are shown. (a) normal group; (b) DSS group; (c) VitD3 group; (d) IFX group (2.5 mg/kg); (e) IFX group (5.0 mg/kg); (f) VitD3+IFX group (2.5 mg/kg); (g) VitD3+IFX group (5.0 mg/kg); (i): IL-10 in splenocyte supernatant was measured by ELISA kit. Data are represented as the mean±standard deviation; n=5 per group; ^$$^*P*<0.01 versus normal group; ^**^ *P*<0.01 versus DSS group; ^##^*P*<0.01 versus IFX group (2.5 mg/kg); ^∆∆^*P*<0.01 versus IFX group (5.0 mg/kg).

IFX: Infliximab; DSS: Dextran sodium sulfate; VitD3: 1,25-dihydroxyvitamin D3; AZA: Azathioprine

Representative immunohistochemical images are shown in the (a) normal group, (b) DSS group, (c) VitD3 group, (d) IFX group (2.5 mg/kg), (e) IFX group (5.0 mg/kg), (f) VitD3+IFX group (2.5 mg/kg), and (g) VitD3+IFX group (5.0 mg/kg), and semiquantitative analysis is shown in (h). (i) The IL-10 level in colonic tissues was measured by an ELISA kit. Data are represented as the mean±standard deviation; n=5 per group; $$P<0.01 versus normal group;**P<0.01 versus DSS group; ##P<0.01 versus IFX group (2.5 mg/kg); ∆∆P<0.01 versus IFX group (5.0 mg/kg).

IFX: Infliximab; DSS: Dextran sodium sulfate; VitD3: 1,25-dihydroxyvitamin D3; Foxp3: Forkhead Box P3

## Discussion

TNF-α plays an important role in the occurrence and development of chronic IBD, and anti-TNF-α therapy can effectively improve disease symptoms (4). However, the loss of response to IFX in some IBD patients remains an important clinical problem. In brief, treatment of IBD by humanized monoclonal antibody directed against TNF-α (IFX) induces complete clinical remission in 33% to 50% of patients, in which some patients do not respond to IFX at the beginning of treatment. In contrast, others respond at the beginning of treatment but lose this response later (6, 7). To overcome resistance to biologics, retrospective data or clinical practices suggest the benefit of the combination of TNF-α inhibitors with immunosuppression. For example, the combined therapy of anti-TNF-α and thiopurines in IBD has been proven to be superior to monotherapy in induction and maintenance of response due to decreased antibody formation against anti-TNF-α agents, increased response rate, and prolonged drug survival (19). However, IFX or thiopurine monotherapy may be associated with more serious adverse events in a dose-dependent manner (20, 21). Previously published work has found that high-dose (HD) IFX may benefit IBD patients who have failed standard doses of IFX, but HD IFX therapy was discontinued in 7.3% of patients for adverse events, and eleven cases of infection required hospitalization for a serious infection rate of 7.41 events per 100 patient-years (20). Moreover, both IFX and thiopurines present powerful immunosuppression, and therefore, the combination will inevitably induce a strong inhibitory effect on the body’s immune system, resulting in serious adverse reactions. It has been shown that the combination of anti-TNF-α agents and thiopurines has been associated in a real-life setting with an increased risk for serious and opportunistic infections compared to monotherapy with both agents (22). Moreover, a recent systematic review with meta-analysis, comprising 261,698 patients, concluded that both anti-TNF-α agents and thiopurines are associated with an increased risk of lymphoma, and the risk associated with combination therapy was significantly higher compared to both unexposed patients and monotherapy (21). Therefore, how to reduce IFX adverse reactions or increase their effects is worthy of further study.

The active vitamin D metabolite VitD3 has a variety of biological activities, including regulation of the immune system (10-11). VitD3 inhibits some parts of adaptative immunity, especially shifting Th1 and Th17 lymphocytes toward a Th2 phenotype, with potential protective effects on some autoimmune diseases (23). Therefore, it has previously been shown that VitD3 has a good effect on UC. In an experimental animal model, VitD3 ameliorated clinical symptoms in an established chronic colitis model (12). For UC patients, vitamin D deficiency is highly prevalent. Patients with longer disease duration, more severe symptoms, and pancolitis are likely to have lower vitamin D levels (24). Vitamin D supplementation was associated with reduced intestinal inflammation in patients with active UC (25). Moreover, vitamin D stimulates innate immunity with potential protective effects against some infectious diseases (26). As a result, VitD3 may be a bilateral immunomodulator rather than a unilateral immunosuppressant. Therefore, the combination of VitD3 with IFX may have advantages in the occurrence of severe adverse events when compared with VitD3 and immunosuppressants. In the present study, we enrolled seven UC patients and found that the median serum level of VitD3 in mild UC patients was significantly higher than that in the severe group. Furthermore, in DSS-induced animals, VitD3 and IFX co-administration synergistically improved symptoms and pathological and hematological manifestations and inhibited intestinal inflammation in colitis mice, and the therapy of the combined group was more effective than that of the corresponding IFX group. Moreover, the other important finding in the present study is that there was no significant difference in the protective effect between IFX (5.0 mg/kg) and VitD3+IFX (2.5 mg/kg) or between VitD3+IFX (5.0 mg/kg) and AZA+IFX (5.0 mg/kg). These results suggest that VitD3 combined with a small dose of IFX may be an alternative therapy for UC patients who are intolerable in response to standard doses of IFX. Moreover, VitD3 combined with a standard dose of IFX may be an alternative therapy for UC patients who are intolerable in response to a combination of IFX and immunosuppressants.

In previous reports, several mechanisms have been proposed to explain the increased effectiveness of combined immunosuppressives and IFX. In brief, the presence of anti-drug antibodies against IFX has been associated with a 4-fold increase in drug clearance, most likely due to enhanced clearance of drug/anti-drug antibody immune complexes. Lower rapid drug clearance results in low or undetectable circulating drug concentrations, which are associated with lower success rates for the induction of remission and with loss of response (9). However, there is limited data to support our present findings. Therefore, further study was conducted to disclose why VitD3 and IFX synergistically repressed the development of colitis. Many studies have revealed the anti-colitis mechanism of VitD3, such as deactivation of the local renin-angiotensin system in the colon, inhibition of NLRP3 inflammasome activation, alteration of the composition of the fecal microbiome, and modulation of T helper (Th)1 and Th17 activation (11, 27-29). We postulated that the addition of VitD3 to IFX therapy may have benefits based on synergy at the pharmacological level. Tregs play a crucial role in the pathogenesis of IBD. Therapeutic arrangement based on Tregs is important to address systemic inflammatory and autoimmune diseases. A decreased number of Tregs was previously observed in patients with IBD compared with healthy controls. Inhibited generation of functionally impaired Tregs contributes to intestinal inflammation, leading to colitis and other complications, while functionally immunosuppressive Tregs have been shown to ameliorate IBD-induced immune responses (17, 30, 31). In a recent study (32), VitD3 was shown to increase Treg profiles and the production of its secreted anti-inflammatory cytokine IL-10. In the present study, VitD3 monotherapy or combination treatment showed more powerful regulation of splenetic Treg differentiation and IL-10 production than IFX alone. Moreover, VitD3 treatment alone or in combination induced increased levels of Foxp3 in colon tissue, which is one of the key transcription factors controlling Treg differentiation.

## Conclusion

In summary, our results demonstrate that VitD3 and IFX synergistically improve colitis in mice. Co-administration of VitD3 with a reduced dose of IFX showed the same protective effect as that of a standard dose of IFX, and administration of VitD3 with a standard dose of IFX showed an identical effect to that of AZA+IFX. Our results suggest that VitD3 used together with IFX is an alternative therapy for UC patients who are intolerable in response to standard doses of IFX or a combination of AZA and IFX. Moreover, VitD3 facilitates the control of colitis by IFX via its powerful regulation of Tregs.
